# Effect of shortened sleep on energy expenditure, core body temperature, and appetite: a human randomised crossover trial

**DOI:** 10.1038/srep39640

**Published:** 2017-01-10

**Authors:** Masanobu Hibi, Chie Kubota, Tomohito Mizuno, Sayaka Aritake, Yuki Mitsui, Mitsuhiro Katashima, Sunao Uchida

**Affiliations:** 1 Health Care Food Research Laboratories, Kao Corporation, Tokyo, Japan; 2 Graduate School of Sport Sciences, Waseda University, Saitama, Japan; 3 Faculty of Sport Sciences, Waseda University, Saitama, Japan

## Abstract

The effects of sleep restriction on energy metabolism and appetite remain controversial. We examined the effects of shortened sleep duration on energy metabolism, core body temperature (CBT), and appetite profiles. Nine healthy men were evaluated in a randomised crossover study under two conditions: a 3.5-h sleep duration and a 7-h sleep duration for three consecutive nights followed by one 7-h recovery sleep night. The subjects’ energy expenditure (EE), substrate utilisation, and CBT were continually measured for 48 h using a whole-room calorimeter. The subjects completed an appetite questionnaire every hour while in the calorimeter. Sleep restriction did not affect total EE or substrate utilisation. The 48-h mean CBT decreased significantly during the 3.5-h sleep condition compared with the 7-h sleep condition (7-h sleep, 36.75 ± 0.11 °C; 3.5-h sleep, 36.68 ± 0.14 °C; p = 0.016). After three consecutive nights of sleep restriction, fasting peptide YY levels and fullness were significantly decreased (p = 0.011), whereas hunger and prospective food consumption were significantly increased, compared to those under the 7-h sleep condition. Shortened sleep increased appetite by decreasing gastric hormone levels, but did not affect EE, suggesting that greater caloric intake during a shortened sleep cycle increases the risk of weight gain.

Obesity is a serious health problem worldwide^1^ and a well-known risk factor for cardiovascular disease, hyperlipidaemia, hypertension, and type 2 diabetes^2^,^3^,^4^. Physical inactivity and/or overeating contribute to the development of obesity^5^, in other words, changes in body weight can be explained by an energy imbalance^6^,^7^. When daily energy intake (EI) surpasses energy expenditure (EE), the energy balance in the body becomes positive. The cumulative effects of even a small daily positive energy balance on body weight regulation are severe^8^. Several epidemiological studies have demonstrated a correlation between insufficient sleep and increased incidence of obesity in adults and children^9^,^10^,^11^,^12^,^13^,^14^. Although sleep disturbances have been recognised to one of the risk factors for obesity^15^,^16^, how sleep curtailment contributes to the physiological and molecular mechanisms by which sleep restriction affects the daily energy balance remains unclear^17^,^18^.

The potential physiological mechanisms related to sleep deprivation and excess energy balance include changes in appetite and increased time to access food, which may influence EI, and extended wakeful time at night, physical inactivity due to fatigue, and decreased thermogenesis, which may affect EE^19^,^20^. Recent intervention studies reported an association between sleep deprivation and hyperphagia. Spiegel *et al*.^21^ demonstrated that 2 days of decreased sleep increases the appetite of healthy young subjects to the same extent as did two key appetite-regulating hormones, the anorexigenic hormone leptin and the orexigenic gut hormone ghrelin. Others reported that shortened sleep leads to greater EI^22^,^23^,^24^,^25^. Yet, these observations have not been reproduced in similar intervention studies^26^,^27^. Namely, increased EI has not been consistently observed following experimental sleep restriction.

The effects of sleep deprivation on energy expenditure are also controversial. Nedeltcheva *et al*.^23^ and St-Onge *et al*.^25^ used double-labelled water under free-living conditions to demonstrate that restricted sleep does not affect 24-h EE. Brondel *et al*.^22^ reported that overnight sleep restriction is followed by a day of increased EI and activity, whereas Bosy-Westphal *et al*.^28^ found no effects of sleep restriction on daytime activity. More recently, some research groups measured 24-h EE and substrate utilisation using whole-room indirect calorimeter^26^,^29^,^30^,^31^. Jung *et al*.^29^ reported that 24-h EE is ~7% higher during periods of total sleep deprivation than during periods of habitual sleep (0-h vs. 8-h sleep time), and Markwald *et al*.^30^ demonstrated that 24-h EE increases ~5% during restricted sleep conditions compared to a normal sleep condition (5-h vs. 9-h sleep time). In the former study, however, EE decreased in the following recovery sleep day, and in the latter study, the increased EE may have been influenced by increased food intake. Moreover, it remains unclear whether the link between shortened sleep time and obesity is a result of changes in homeostatic consumption behaviour or a decrease in EE.

The aim of the present study was to determine whether shortened sleep (3.5-h/night for 3 nights) affects energy metabolism, core body temperature (CBT), and appetite, thereby altering the energy balance in normal-weight healthy young men. We hypothesised that compared to normal sleep duration, a shorter sleep duration would lead to greater appetite sensations and decreased EE.

## Results

### Baseline characteristics and sleep variables

Nine subjects completed the trial according to the study schedule (mean ± SD; age, 23 ± 2 y; body mass index, 22.2 ± 3.0 kg/m^2^). Throughout the 1-week pre-laboratory period, the mean sleep time, as determined by wrist-actigraphy, was 460 ± 39 min/night. The mean total sleep time in the calorimeter based on polysomnography was 408.3 ± 7.2 min/night during the 7-h sleep condition and 206.7 ± 2.7 min/night during the 3.5-h sleep condition (day 4 night). During the recovery night, the sleep time did not differ significantly between subjects from either sleep condition (7-h sleep, 407.7 ± 7.2 min/night; 3.5-h sleep, 412.8 ± 4.9 min/night). Although the sleep onset latency was significantly shorter in the 3.5-h sleep condition than in the 7-h sleep condition (day 4 night: 7-h sleep; 12.8 ± 6.2 min/night; 3.5-h sleep; 5.4 ± 2.6 min/night, p = 0.007), the effect of the sleep onset latency during the recovery sleep (day 5 night) was also shorter, but not significantly different between sleep conditions (7-h sleep, 14.7 ± 5.5 min; 3.5-h sleep, 10.9 ± 6.0 min, p = 0.151). The duration of slow wave sleep on day 4 night did not differ significantly between sleep conditions (7-h sleep, 66.7 ± 31.5 min/night; 3.5-h sleep, 79.7 ± 34.1 min/night, p = 0.113), and the duration of slow wave sleep on the recovery sleep night (day 5) did not differ significantly between conditions (7-h sleep, 78.4 ± 24.1 min/night; 3.5-h sleep, 89.8 ± 34.1 min/night, p = 0.170). The duration of rapid eye movement (REM) sleep did not differ significantly among the shortened sleep night, recovery sleep night, and normal sleep night.

### Energy expenditure and substrate oxidation

**Figure 1:**
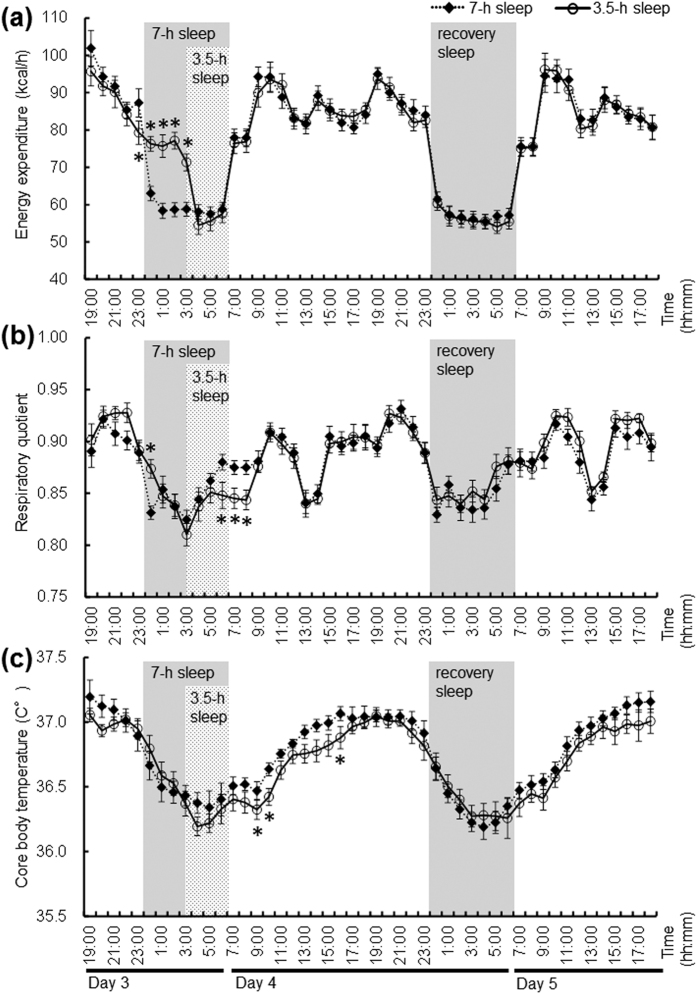
Hourly energy expenditure (EE) (**a**) (n = 9), respiratory quotient (RQ) (**b**) (n = 9), and core body temperature (CBT) (**c**) (n = 7) during 48 h in the whole-room indirect calorimeter. Data are expressed as the mean ± SD value per hour. The black diamonds with broken lines represent the 7-h sleep condition and the white circles with black lines represent the 3.5-h sleep condition. A repeated-measures ANOVA revealed that EE over 48 h varied with the sleep condition and a time, as demonstrated by the non-significant effect of sleep condition (p = 0.705), but there was a significant effect of time (p < 0.001) and a time × condition interaction (p < 0.001). The hourly RQ across 48 h varied with sleep condition and time as demonstrated by the nonsignificant effect of condition (p = 0.442) and condition × time interaction (p = 0.317), with a significant effect of time (p < 0.001). A repeated-measures ANOVA revealed that CBT varied with sleep condition and time over 48 h, as demonstrated by a significant effect of condition (p < 0.001) and time (p < 0.001); there was no significant condition x time interaction (p = 0.571). *Significantly different from the 7-h sleep condition (p < 0.05, after Bonferroni’s correction).

The 48-h total energy expenditure (TEE), and the 24-h TEE on day 3/4 and on the recovery night of day 4/5 did not differ significantly between the 7-h and 3.5-h sleep conditions (Table 1). The hourly EE over 48 h varied with sleep condition and time, as demonstrated by the lack of a significant effect of sleep condition, and the presence of a significant effect of time (p < 0.001) and the condition x time interaction (p < 0.001; Fig. 1a). Night-time EE on day 3 (00:00 to 07:00) was higher in the 3.5-h sleep condition than in the 7-h sleep condition (7-h sleep, 409 ± 37 kcal/d; 3.5-h sleep, 464 ± 45 kcal/d, p < 0.001). The 24-h energy balance measured using the calorimeter on day 3/4 and 4/5 was slightly positive, but did not differ between sleep conditions (7-h sleep, 114 ± 73 kcal/d and 145 ±  89 kcal/d, respectivly; 3.5-h sleep, 77 ± 72 kcal/d and 157 ± 62 kcal/d, respectively). The 48-h respiratory quotient (RQ) values, and the 24-h RQ on day 3/4 and day 4/5 did not differ between sleep conditions (Table 1). The hourly RQ over 48 h varied with sleep condition and time, as demonstrated by the lack of a significant effect of condition, the condition x time interaction, and the presence of a significant effect of time (p < 0.001; Fig. 1b). The 48-h average activity (%) and the 24-h activity on the recovery night of day 4/5 did not differ significantly between the 7-h and 3.5-h sleep conditions (Table 1). The 24-h average activity on day 3/4 was significantly higher in the 3.5-h sleep condition than in the 7-h sleep condition (p = 0.016).

### Core body temperature

Due to technical issues, CBT data were acquired for only seven subjects. The mean CBT over 48 h was significantly higher in the 7-h sleep condition than in the 3.5-h sleep condition (Table 1). The 24-h mean CBT values for day 3/4 and 4/5 did not differ significantly between sleep condition (Table 1), but after 3 days of restricted sleep, mean CBT was significantly lower in the 3.5-h sleep condition than in the 7-h sleep condition on day 4 (7-h sleep, 36.72 ± 0.12 °C; 3.5-h sleep, 36.65 ± 0.15 °C, p = 0.015). The CBT profiles over 48 h varied with sleep condition and time, as demonstrated by the significant effect of condition (p < 0.001) and time (p < 0.001), but the lack of a significant condition x time interaction (Fig. 1c).

### Appetite questionnaire

**Figure 2:**
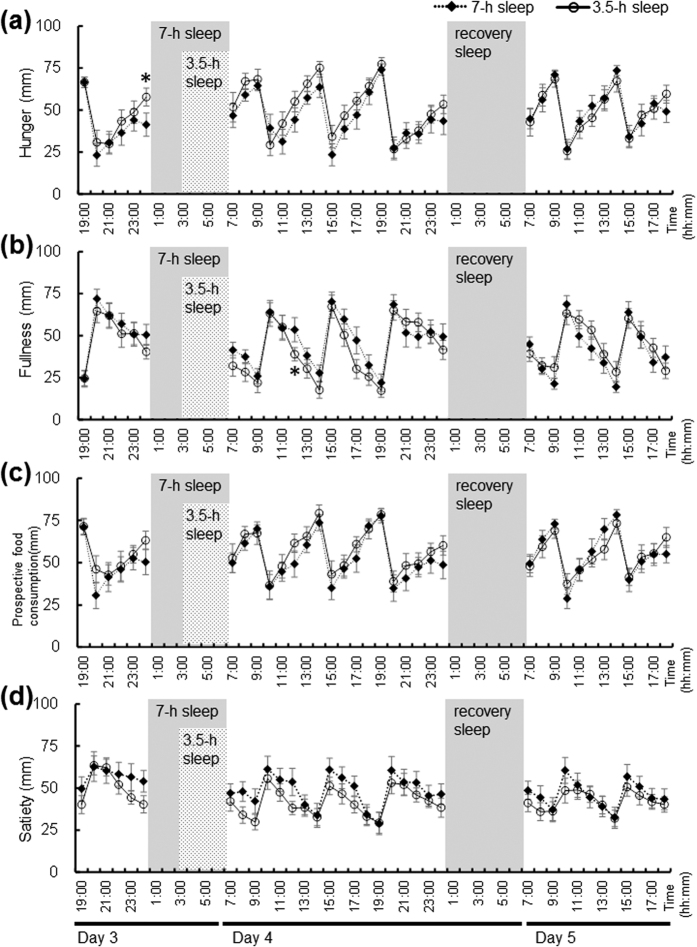
Mean appetite ratings of hunger (**a**), fullness (**b**), prospective food consumption (**c**), and satiety (**d**) in 3.5-h and 7-h sleep conditions after 48 h in the whole-room indirect calorimeter. The data are expressed as the mean ± SD appetite ratings (n = 9). The black diamonds represent the 7-h sleep condition, and the white circles represent the 3.5-h sleep condition. An ANOVA revealed a significant effect of condition and time for hunger (p = 0.004 and p < 0.001), but there was no significant condition x time interaction (p = 0.857). There was a significant effect of time for fullness (p < 0.001), but there was no significant effect of condition and condition x time interaction (p = 0.064 and p = 0.522). There was a significant effect of time for prospective food consumption (p < 0.001), but there was no significant effect of condition and condition x time interaction (p = 0.284 and p = 0.833). There was a significant effect of time for satiety (p < 0.001), but there was no significant effect of condition and condition x time interaction (p = 0.094 and p = 0.758). *Significantly different from 7-h sleep condition (p < 0.05, after Bonferroni’s correction).

The area under the curves (AUCs) for the hungry and prospective to food consumption from visual analogue scale (VAS) scores were significantly increased, and the fullness score was significantly decreased over 24 h during the 3.5-h sleep condition on day 3/4 compared to the 7-h sleep condition (Table 2). The AUCs for the 24 h appetite profiles, however, did not differ significantly between the 3.5-h sleep condition and the 7-h sleep condition over 24 h on day 4/5 during the recovery night. Profiles of mean appetite values (i.e., hunger, fullness, prospective food consumption, and satiety) based on hourly VAS questionnaires are shown in Fig. 2. The condition x time effects for hunger, fullness, prospective food consumption, and satiety were not significantly different between sleep conditions. The main effects of sleep condition on fullness, prospective food consumption, and satiety were not significantly different, but the effect of sleep condition on hunger was significant (p = 0.004).

### Fasting blood and urine analysis

**Figure 3:**
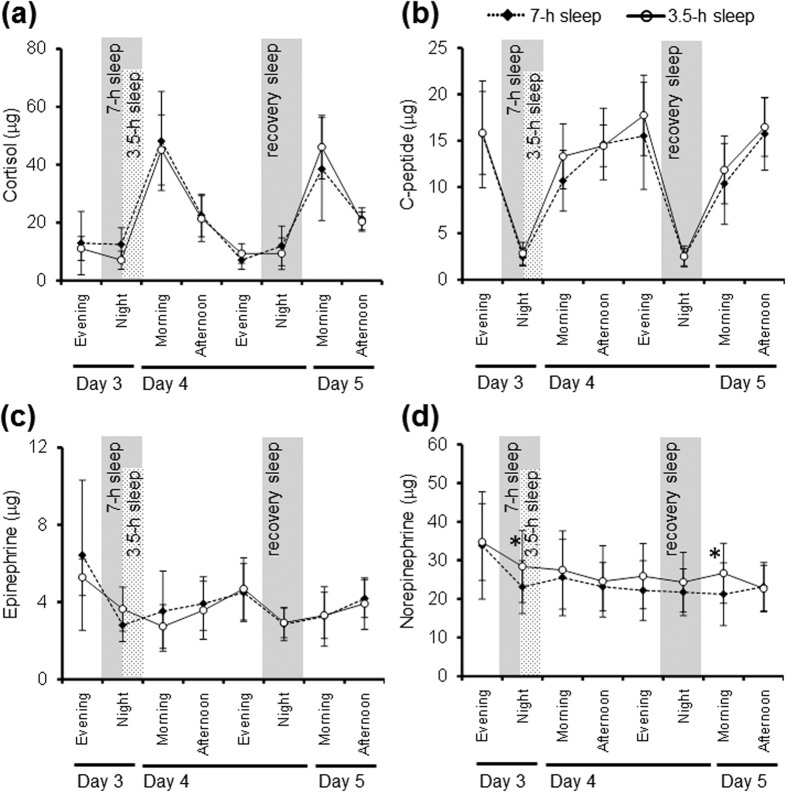
Mean urinary metabolite levels of cortisol (**a**), c-peptides (**b**), epinephrine (**c**), and norepinephrine (**d**) during the 3.5-h and 7-h sleep conditions after 48 h in the whole-room indirect calorimeter. The data are expressed as the mean ± SD values (n = 9). The black diamonds represent the 7-h sleep condition and the white circle represent the 3.5-h sleep condition. An ANOVA revealed a significant effect of time for epinephrine, cortisol, and c-peptides (p < 0.001), but there was no significant effect of condition or condition x time interaction. There was a significant effect of time and condition for norepinephrine (p < 0.001 and p = 0.006, respectively), but there was no significant effect of the condition x time interaction (p = 0.725). *Significantly different from the 7-h sleep condition (p < 0.05, after Bonferroni’s correction).

Fasting blood lipid and hormone values after the 3-night sleep restriction and recovery sleep are shown in Table 3. Plasma peptide YY (PYY) concentrations were significantly lower after the 3-night sleep restriction in the 3.5-h sleep condition than in the 7-h sleep condition (p = 0.011). Plasma glucagon-like peptide-1 (GLP-1) levels after the 3-night sleep restriction tended to be lower in the 3.5-h sleep condition than in the 7-h sleep condition (p = 0.055). Tri-iodothyronine and thyroxin, high-density lipoprotein-cholesterol, low-density lipoprotein-cholesterol, triacylglycerol, non-esterified fatty acids, adiponectin, cortisol, and leptin levels did not differ significantly between sleep conditions. The mean urinary metabolite profiles (cortisol, c-peptides, epinephrine, and norepinephrine) are shown in Fig. 3. Condition x time effects were not detected for epinephrine, norepinephrine, cortisol, or c-peptide levels. The main effects of sleep condition on epinephrine, cortisol, and c-peptide levels were not significant, but there was a significant main effect of sleep conditions on norepinephrine (p = 0.006). After Bonferroni’s correction for multiple comparisons, norepinephrine levels were significantly higher in the 3.5-h sleep condition during the hours between 00:00–07:00 on day 3/4 and between 07:00–14:00 on day 5 than in the 7-h sleep condition.

## Discussion

The present study investigated the effects of three consecutive nights of reduced sleep duration (3.5-h sleep vs. 7-h sleep) on energy metabolism in healthy young men based on whole-room indirect calorimeter, CBT by rectal core body temperature thermometry, appetite profiles with hourly VAS questionnaires, and blood gut hormone levels. Our results indicated that shortened sleep for 3 nights did not decrease TEE levels, although the mean CBT was significantly decreased during the 3.5-h sleep condition compared to the 7-h sleep condition. Furthermore, in the 3.5-h sleep condition, hunger and prospective food consumption scores increased, whereas the fullness score decreased, and fasting levels of the anorexigenic gut hormones PYY and GLP-1 also decreased. These findings indicate that insufficient sleep increases food intake, potentially associated with changes in PYY and GLP-1 levels, which could lead to weight gain. Moreover, reduced CBT and increased urine norepinephrine levels in the 3.5-h sleep condition might indicate a sleep deprivation-induced disruption of the circadian rhythm of body temperature regulation in association with whole-body EE rhythm.

We demonstrated that the TEE values during 3.5-h sleep condition did not differ significantly from those during the 7-h sleep condition. Nevertheless, we observed a significant effect of sleep deprivation on hourly EE patterns over 48 h (p < 0.001), and an approximately 55 kcal increase in the night-time EE on the night of day 3 during the 3.5-h sleep condition compared to the 7-h sleep condition. These changes in EE were likely due to an increase in EE during the night hours spent awake. Markwald *et al*.^30^, Klingenberg *et al*.^26^, and Shechter *et al*.^31^ reported that EE changes at night. Whole-room calorimeter, which is the gold standard for measuring energy metabolism, shows an ~5% increase in 24-h EE during restricted sleep conditions compared to habitual sleep in healthy adults^26^,^30^ and in healthy women^31^. In our study, shortened sleep led to an ~2% increase in the 24-h TEE, although this increase was not statistically significant. Despite the increase in night-time EE, TEE did not change in the present study, in contrast to the findings from the other studies. Furthermore, the hourly EE after one night of recovery sleep did not differ between the sleep conditions. The short-term (~3 days) partial sleep restriction did not affect EE after one night of recovery sleep. Jung *et al*.^29^ reported that 24-h TEE increases by ~7% during total sleep deprivation (awake for 40-h) and decreases ~5% during the recovery condition compared to baseline. They also reported that the 7% increase in 24-h TEE on the total sleep deprivation day is nearly offset by the energy saved during the recovery day, resulting in a net cost of 2% across the 48 h examined^29^. Although the sleep deprivation conditions differed between the studies (total sleep deprivation for 1 day vs. shortened sleep for 3 days), our data are consistent with those from Jung *et al*.^29^. Thus, the lack of an effect on EE after sleep restriction suggests that EE does not contribute to the potential weight gain reported in the epidemiological studies^9^,^10^,^11^,^12^,^13^,^14^.

The 48-h mean CBT and the 24-h mean CBT significantly decreased by 0.07 °C during the 3.5-h sleep condition compared to that observed during the 7-h sleep condition. The close relationship between the metabolic rate and CBT^32^ may partly explain the risk of future weight gain. A 24-h mean decrease in the CBT of 0.07 °C would account for a body fat accumulation of 0.5 kg per year^33^,^34^, which is a relatively small amount that may change according to lifestyle. Few studies have directly addressed the relationship between shortened sleep and lower CBT^20^. Bach *et al*.^35^ reported a decrease in the daytime CBT over 5 nights of 4-h sleep restriction compared to baseline values. Benedict *et al*.^36^ observed higher night-time CBT during 24-h total sleep deprivation (~+0.2 °C) and lower daytime CBT during the following day (~−0.1 °C). Our findings are partly consistent with those of the previous reports, suggesting a disruption in the circadian rhythm of thermoregulation. We also observed higher norepinephrine levels during the 3.5-h sleep condition and the following recovery night. Previous literature and the data obtained in the present study might support the idea that lowering the CBT affects norepinephrine release. Frank *et al*.^37^ reported a significant increase in the norepinephrine concentration when CBT was lowered (36.0 °C) in younger subjects. Faraut *et al*.^38^ observed a 2.5-fold increase in norepinephrine levels during the day after a sleep-restricted night, but no change in epinephrine or dopamine levels. Short or restricted sleep times have also been reported to impair neural brain activity^39^. The circadian rhythms of CBT and thermoregulation are regulated by the hypothalamus, and sleep restriction potentially affects hypothalamic function, resulting in dysfunctional control of body temperature^40^. Further studies must be conducted to clarify the association among sleep restriction, circadian rhythms, and related brain function.

Our findings indicated that the 24-h RQ did not differ significantly between the 7-h and 3.5-h sleep conditions. Other researchers examined 24-h substrate utilisation measured using whole-room indirect calorimetry under sedentary conditions^26^,^29^,^30^,^31^,^41^. Shechter *et al*.^31^ reported that the 24-h RQ after 3 nights of short (4-h/night) versus habitual (8-h/night) sleep duration dose not differ significantly under fixed meal conditions. Our findings are consistent with these findings in that substrate utilisation did not differ after sleep restriction despite the greater number of hours spent awake in the 3.5-h sleep condition. Sleep restriction is reported to increase the insulin response^23^, but in the present study, the 24-h levels of urinary C-peptide and cortisol were not affected by shortened sleep time, which is in good agreement with the substrate utilisation values in both sleep conditions. These data do not support the concept that sleep restriction alters substrate utilisation in such a way as to favour future weight gain. Similar to our findings that sleep restriction significantly decreased fasting RQ, Shechter *et al*.^41^ and Klingenberg *et al*.^26^ reported a lower fasting RQ after shortened versus habitual sleep. The temporarily decreased RQ, which might be affected by temporary differences in energy balance from the prolonged wakefulness in shortened sleep conditions, was not reflected by the 24-h RQ.

In the present study, sleep loss led to a significant increase in the hunger and the prospective food consumption score, and a significant decrease in the fullness score during the 3.5-h sleep condition. Moreover, fasting PYY concentrations were significantly lower, and fasting GLP-1 concentrations tended to be lower after the 3-night sleep restriction. Spiegel *et al*.^21^ and Benedict *et al*.^36^ also reported increased hunger feelings after two nights of short sleep duration or a total of one night of sleep deprivation relative to habitual sleep. Moreover, sleep fragmentation reduced daily GLP-1 profiles and the fullness score^42^. PYY received special attention in clinical studies after Batterham *et al*.^43^ demonstrated that infusions of PYY in doses mimicking postprandial increases in plasma PYY levels reduces appetite and food intake for 12- to 24-h in both normal-weight and obese subjects. Our findings should be taken into account with these previous findings that the gut anorexigenic hormone levels are related to feelings of hunger. In addition, decreased sleep in healthy subjects increased the consumption of snacks, particularly at night^23^. Our results identified differences in appetite profiles based on night-time sleep duration. It is not clear why shortened sleep did not affect glucose or insulin levels. Further investigation is required to elucidate this matter.

A limitation of the present study was that the ‘normal’ sleep condition of 7 h may be considered a mild sleep disturbance compared to the studies providing 8 or 9-h sleep opportunities. The Japan Collaborative Cohort Study reported a U-shaped relationship between sleep duration and total mortality, with a nadir at a 7-h sleep duration, in a large-scale prospective study of Japanese individuals^44^. Moreover, the clinical significance of a change in mean 48-h CBT of 0.1 degrees or less must be further investigated. Additional potential limitations are the small sample size, the male-only subject population, and the fact that actual food intake, postprandial blood samples, and ghrelin levels were not assessed. We did not collect and analyse the subjects’ sleep patterns during the washout period or the eating behaviour of the subjects during the pre-intervention period and washout period, which could affect the second intervention and outcomes. Therefore, more research with a larger sample size including women is necessary to establish general observations. Last, because of the limited space for physical activity in the calorimeter, and because the subjects were not allowed to sleep during the day, our results only partially resemble daily life.

In conclusion, the present findings revealed that sleep restriction reduced gut hormones (PYY and GLP-1) and increased appetite sensations, but did not alter EE or substrate utilisation during 48-h calorimeter measurements. Moreover, a 3-night shortened sleep intervention reduced CBT for 48 h in healthy young men. Three nights of short sleep duration might lead to a positive energy balance. These findings suggest that the quantity of sleep-time leads to changes in individual energy balance and circadian rhythms and may increase the risk of obesity.

## Methods

### Subjects

The study was approved by the Ethics Committee of Waseda University, in accordance with the approved ethics guidelines. This trial was registered with the University hospital medical information network (UMIN) clinical trials registry (http://www.umin.ac.jp/) on December 6, 2013 as UMIN000012506. All of the participants provided written informed consent before study commencement. Nine healthy young men participated in the study (mean ± SD, age 23 ± 2 y; body mass index 22.2 ± 3.0 kg/m^2^). We calculated the minimum number of subjects to be enrolled under the empirical, preliminary assumption of a mean difference of 90 kcal/d with a standard deviation of 90 kcal/d in 24-h EE between the sleep conditions. Based on the sample size calculation, 9 subjects would be required to detect a significant difference with a two-sided paired t-test with 75% power and a 5% alpha level. The subjects were recruited through poster advertisements. The inclusion criteria were as follows: 20 to 40 y of age, body mass index of 18.0 to 29.9 (kg/m^2^), and a normal sleep pattern. The exclusion criteria were as follows: self-reported sleep problems (Pittsburgh Sleep Quality Index score >10); shift-work; smoking; excessive alcohol intake (>30 g alcohol/day); history of, or currently taking medication for cardiovascular disease, hypertension, diabetes, hypercholesterolaemia, hyperglycaemia, or hyperlipidaemia; and the use of prescription medications affecting sleep or metabolism.

### Experimental design

**Figure 4:**
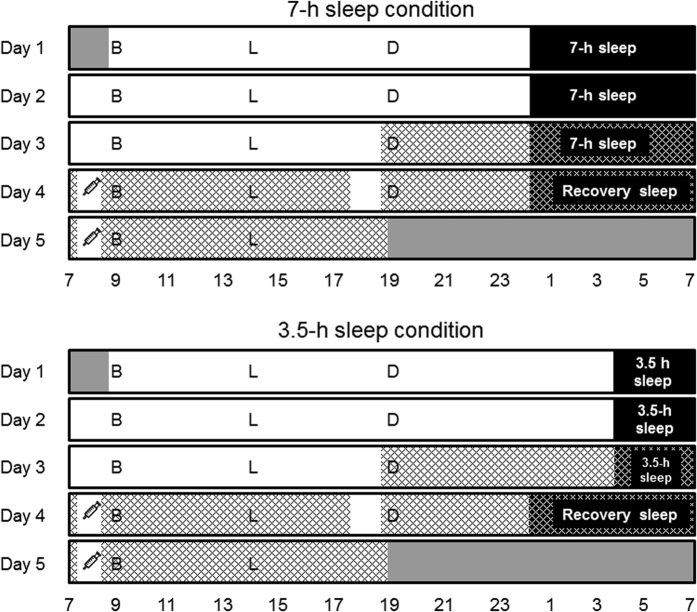
Illustration of the study scheme. The participants spent awake and sleeping time in the laboratory from 09:00 on day 1 to 19:00 on day 5. The figure shows the time spent in and out of the respiratory chamber to remove or install the polysomnography device and to take a shower (removed from 07:15 to 07:45 on days 4 and 5 and installed from 17:15 to 18:15 on day 4). All meals were given at the same time in both conditions. The 7-h sleeping times on days 1 to 3 and the recovery sleep on day 4 and the 3.5-h sleeping time on days 1 to 3 are shown (black). The VAS questionnaires were provided every hour. Overnight polysomnography was performed to examine the night-time sleep quality on days 3 and 4. Core body temperatures were continuously measured from 19:00 on day 3 to 19:00 on day 5, except for when subjects were taking a shower. Key: grey areas – out of the study protocol, hatched areas – inside the respiratory chamber, black areas – time in bed. B, breakfast at 09:00; L, lunch at 14:00; D, dinner at 19:00; and syringe symbols represent blood drawing at 07:30.

This was a randomised crossover study that included one acclimatisation day and two 5-day intervention periods with either a 7-h sleep condition or a 3.5-h sleep condition (Fig. 4). The 7-h sleep condition involved a 7-h sleep opportunity (from 00:00 to 07:00) for three consecutive nights and a 7-h sleep opportunity for one recovery night (from 00:00 to 07:00). The 3.5-h sleep condition comprised 3.5-h sleep opportunities (from 03:30 to 07:00) for 3 consecutive nights and a 7-h sleep opportunity for one recovery night (from 00:00 to 07:00). A wash-out period of approximately 2 weeks was inserted between interventions. On the acclimatisation day, the subjects slept from 00:00 to 07:00 in whole-room indirect calorimeters at the Kao Health Care Food Research Laboratories (Tokyo, Japan) with polysomnographic recordings. The meals provided in the laboratory were based on energy requirements estimated by the basal metabolic rate (BMR) equation^45^ with a physical activity level of 1.6. Meal composition was 15 per cent of energy (E%) protein, 25 E% fat, and 60 E% carbohydrate, and both sleep condition groups received meals of identical quantity and composition. The calories were distributed among meals as follows: 30 E% breakfast, 30 E% lunch, and 40 E% dinner. Dieticians provided breakfast, lunch, and dinner at 09:00, 14:00, and 19:00, respectively. In the sleep condition, the subjects were required to go to bed and room lights were turned off at 00:00 for the 7-h bedtime and at 03:30 for the 3.5-h bedtime. The subjects were awakened at 07:00. The investigators continuously monitored wakefulness and compliance with the protocol. The subjects exited the calorimeter and removed the polysomnographic device at 07:15 and put it back on at 07:45 on days 4 and 5. The subjects exited the calorimeter at 17:15 and re-entered at 18:15 on day 4. After exiting the calorimeter, the participants were allowed to shower. We fed the subjects a eucaloric diet, which was based on their predicted BMR multiplied by a physical activity level of 1.3 to account for their decreased physical activity due to the limited space in the calorimeter (1.3 × BMR, between chamber stays). Meal compositions and caloric distributions were consistent with those outside of the calorimeter. On days 4 and 5, we obtained a fasting blood sample from each subject at 07:30. During the wash-out period, we did not restrict food intake or exercise and encouraged the participants to maintain their normal lifestyles.

### Sleep and core body temperature recording

Sleep was recorded using polysomnography (Polymate AP216, TEAC Corp., Tokyo, Japan) as four electroencephalograms with two reference electrodes in the earlobes, two electrooculograms, two submental electromyograms, and two electrocardiograms recordings. The epoch lengths for visual and computer analyses were 30 s. The recordings of REM and non-REM sleep were analysed using fast Fourier transformation. We calculated total power as the sum of each frequency band power from all epochs. CBT was recorded using a portable device (LT-8A, Gram Corporation, Saitama, Japan). Each subject inserted a probe into their rectum, and the probe remained there except when they took a shower or defecated. Temperature was continuously recorded every 1 min for 48 h. All recorded data were analysed except for the excessively low temperature points, 2 h after a shower, and 45 min after waking up. The hourly averaged data were calculated across 60-min periods to obtain a mean CBT for the transient response analysis.

### Appetite questionnaire

Appetite profiles (hunger, fullness, prospective food consumption, and satiety) were measured using the 100-mm VAS questionnaire^46^, which was translated into Japanese from English^47^. We measured hourly appetite profiles 37 times between the awake time from 19:00 on day 3 to 19:00 on day 5. The 24-h appetite scores were calculated as the AUCs on day 3/4 (from 19:00 on day 3 to 19:00 on day 4) and day 4/5 (from 19:00 on day 4 to 19:00 on day 5).

### Whole-room indirect calorimeter measurements

EE and substrate utilisation for each subject were measured in the respiratory chamber over 48 h in both conditions. Whole-room indirect calorimeter measurements were obtained by the previously described methods^48^. In brief, room temperature, humidity, and fresh airflow were set to 25 °C, 50%, and 70 L/min, respectively. Oxygen consumption (VO_2_) and carbon dioxide production (VCO_2_) were calculated using the method reported by Henning *et al*.^49^ VO_2_ and VCO_2_ were calculated across 60-min period to obtain EE and RQ values for the transient response analysis. TEE and RQ values were determined based on the 24-h VO_2_ and VCO_2_ values^50^,^51^. TEE and RQ were also calculated for three different periods: day3/4 (from 19:00 of day 3 to 19:00 of day 4), day4/5 (from 19:00 of day 4 to 19:00 of day 5), and day 4 (from 07:00 of day 4 to 07:00 of day 5). We calculated energy balance by subtracting TEE from actual EI for each 24 h periods. We monitored activity levels in the calorimeter using an infrared motion sensor (Matsushita Automation Controls Co, Ltd, AMP2009B01, Tokyo, Japan). A digital balance, accurate to 0.01 kg (CQ100LW, Ohaus Corp., Pine Brook, NJ), was used to measure body weight prior to the subject entering the calorimeter

### Blood sample analysis

We collected a fasting blood samples from each subject after he exited the respiratory chamber at 07:30 on days 4 and 5. Serum triacylglycerol, non-esterified fatty acid, and glucose levels were measured using standard enzymatic techniques. Serum low-density lipoprotein-cholesterol and high-density lipoprotein-cholesterol concentrations were measured using standard direct methods. Thyroid-stimulating hormone, tri-iodothyronine, and thyroxin were assayed using an electrochemiluminescence immunoassay method. Serum insulin, adiponectin, and GLP-1 concentrations were assayed using an immunoenzymatic method. Serum leptin and cortisol concentrations were determined using a radio-immunoassay method. All of the measurements were performed by LSI Medience Corporation (Tokyo, Japan). Plasma PYY concentrations were determined using an ELISA kit (Millipore, Billerica, MA).

### Urinary analysis

All of the urine samples were collected and weighed while the subjects were in the calorimeter. The subjects were instructed to store urine samples during the following time points: 19:00–24:00, 24:00–07:00, 07:00–14:00, and 14:00–19:00. Urinary cortisol and c-peptide were determined by radioimmunoassay after extraction with dichloromethane. Urinary excretion of norepinephrine and epinephrine were measured by high-pressure liquid chromatography with a cation exchange column, separated by reversed-phase chromatography (model no. 126; Beckman Instruments, San Ramon, CA), and detected with an electrochemical detector (model LC-4B; BioAnalytical Systems, West Lafayette, IN).

### Statistical analysis

The data are presented as the means ± SD unless otherwise indicated. All crossover data for both treatments were compared using paired *t*-tests (two-sided *α* = 0.05). A mixed-model repeated-measures analysis of variance (ANOVA) was used to assess the significance of differences in the profiles of EE, RQ, CBT, and appetite profiles for hourly data with the main effects of condition, time, and condition x time interaction as fixed effects. In addition, a mixed-model ANOVA was used to assess significant changes in the urine metabolite values for each time period. Bonferonni’s correction for multiple comparisons was used to correct for the number of planned comparisons. Statistical analyses were performed using SPSS statistical software (Version 19, IBM Japan, Ltd., Tokyo, Japan).

## Additional Information

**How to cite this article**: Hibi, M. *et al*. Effect of shortened sleep on energy expenditure, core body temperature, and appetite: a human randomised crossover trial. *Sci. Rep.* **7**, 39640; doi: 10.1038/srep39640 (2017).

**Publisher's note:** Springer Nature remains neutral with regard to jurisdictional claims in published maps and institutional affiliations.

## Figures and Tables

**Table 1:** Twenty-four-hour EE, RQ, CBT, and activity for the 48-h stay in the calorimeter during the 3.5-h sleep condition and 7-h sleep condition^1^.

		7-h sleep	3.5-h sleep	*P* value^2^
EE (kcal/d)	Day 3/4^3^	1874 ± 145	1911 ± 155	0.183
	Day 4/5^4^	1844 ± 151	1831 ± 149	0.523
	48-h^5^	3717 ± 288	3741 ± 303	0.508
RQ	Day 3/4	0.881 ± 0.016	0.878 ± 0.021	0.656
	Day 4/5	0.884 ± 0.017	0.891 ± 0.012	0.121
	48-h	0.883 ± 0.015	0.885 ± 0.015	0.519
CBT (°C)	Day 3/4	36.76 ± 0.15	36.67 ± 0.13	0.122
	Day 4/5	36.74 ± 0.13	36.69 ± 0.16	0.116
	48-h	36.75 ± 0.12	36.68 ± 0.14	0.016
Activity (%)	Day 3/4	22.8 ± 3.9	27.3 ± 6.6	0.016
	Day 4/5	22.3 ± 6.0	23.3 ± 5.2	0.483
	48-h	22.6 ± 4.8	25.1 ± 5.5	0.062

^1^EE, RQ, and activity data are expressed as mean ± SD; n = 9. CBT data are expressed as mean ± SD; n = 7. CBT, core body temperature; RQ, respiratory quotient; EE, energy expenditure.

^2^*P* values are calculated by a paired *t* test.

^3^Time period on day 3/4 is from 19:00 of day 3 to 19:00 of day 4.

^4^Time period on day 4/5 is from 19:00 of day 4 to 19:00 of day 5.

^5^Time period a total of 48 h is from 19:00 of day 3 to 19:00 of day 5.

**Table 2:** Twenty-four hour appetite scores calculated as the area under the curve during day 3/4 and 4/5 of the intervention with 3.5-h sleep or 7-h sleep^1^.

		7-h sleep	3.5-h sleep	*P* value 2
Hunger (mm·h)	Day 3/4^3^	774 ± 217	883 ± 177	0.021
	Day 4/5^4^	779 ± 218	784 ± 176	0.879
Fullness (mm·h)	Day 3/4	822 ± 193	716 ± 141	0.020
	Day 4/5	734 ± 152	771 ± 180	0.224
Prospective food consumption (mm·h)	Day 3/4	896 ± 232	973 ± 203	0.035
	Day 4/5	874 ± 228	896 ± 205	0.343
Satiety (mm·h)	Day 3/4	864 ± 296	745 ± 187	0.073
	Day 4/5	780 ± 271	713 ± 207	0.189

^1^All data are expressed as the mean ± SD; n = 9.

^2^*P* values are calculated by paired *t* tests.

^3^Time period on day 3/4 is from 19:00 of day 3 to 19:00 of day 4.

^4^Time period on day 4/5 is from 19:00 of day 4 to 19:00 of day 5.

**Table 3:** Fasting blood metabolites after the 3-day sleep restriction or one recovery night^1^.

	Day 4^3^			Day 5^4^		
	7-h sleep	3.5-h sleep	*P*^2^	7-h sleep	3.5-h sleep	*P*
Glucose (mg/dL)	88.8 ± 4.6	87.3 ± 3.6	0.311	88.9 ± 3.6	88.9 ± 4.2	1.000
Insulin (μU/mL)	5.02 ± 1.85	4.52 ± 1.13	0.480	4.89 ± 2.32	5.62 ± 1.63	0.436
TG (mg/dL)	108 ± 28	98 ± 24	0.102	106 ± 32	101 ± 29	0.362
HDL-C (mg/dL)	53 ± 7	56 ± 11	0.122	52 ± 7	55 ± 10	0.145
LDL-C (mg/dL)	109 ± 29	104 ± 17	0.402	110 ± 31	106 ± 19	0.464
NEFA (mEq/L)	0.342 ± 0.100	0.416 ± 0.097	0.109	0.339 ± 0.111	0.304 ± 0.112	0.196
T3 (ng/dL)	102.9 ± 14.9	101.7 ± 11.1	0.723	95.0 ± 8.3	94.4 ± 10.0	0.516
T4 (μg/dL)	8.1 ± 0.9	8.2 ± 0.8	0.464	8.3 ± 0.7	8.3 ± 0.8	0.946
TSH (μIU/mL)	1.8 ± 1.1	2.0 ± 1.0	0.071	1.7 ± 0.9	1.5 ± 0.7	0.034
Leptin (ng/mL)	4.7 ± 2.0	4.5 ± 1.8	0.680	4.8 ± 2.1	4.4 ± 2.2	0.172
Adiponectin (μg/L)	8.9 ± 3.6	8.9 ± 4.0	0.862	8.6 ± 3.7	8.7 ± 4.0	0.667
GLP-1 (pmol/L)	2.1 ± 1.0	1.4 ± 0.6	0.055	1.6 ± 0.8	1.4 ± 0.5	0.260
PYY (ng/mL)	186.2 ± 34.8	163.0 ± 45.5	0.011	185.0 ± 36.7	179.9 ± 29.2	0.541
Cortisol (μg/dL)	17.8 ± 1.8	18.3 ± 2.1	0.505	19.5 ± 1.7	19.2 ± 1.8	0.604

^1^All data are expressed as the mean ± SD; n = 9. GLP-1, glucagon-like peptide-1; HDL-C, high density lipoprotein cholesterol; LDL-C, low density lipoprotein cholesterol; NEFA, non-esterified fatty acid; PYY, peptide YY; T3, tri-iodothyronine; T4, thyroxin; TG, triglyceride; TSH, thyroid stimulating hormone.

^2^P values are calculated by paired *t* tests.

^3^Time point on Day 4 is 07:30 on day 4 after the 3- day sleep restriction.

^4^Time point on Day 5 is 07:30 on day 5 after the recovery sleep.
